# Prospective randomized feasibility trial to assess the use of rhPDGF-BB in treatment of distal radius fractures

**DOI:** 10.1186/s13018-015-0174-z

**Published:** 2015-03-21

**Authors:** Albert Christersson, Bengt Sandén, Sune Larsson

**Affiliations:** Department of Orthopedics, Uppsala University, Sjukhusvägen 1, 751 85 Uppsala, Sweden

**Keywords:** Distal radius fracture, External fixation, PDGF, Calcium phosphate, Radiographic evaluation, Clinical evaluation, Prospective, Randomized

## Abstract

**Background:**

Recombinant human platelet-derived growth factor BB (rhPDGF-BB) combined with an osteoconductive scaffold (β-TCP) has been demonstrated to increase bone formation, but rhPDGF-BB has not been studied in human fractures. The purpose of this study was to evaluate the safety and potential use of locally administered rhPDGF-BB/β-TCP (Augment®) in acute wrist fractures.

**Methods:**

Forty patients with unstable distal radial fracture were randomized to closed reduction and external fixation alone (*n* = 20) or combined with injection of rhPDGF-BB/β-TCP (Augment®) into the fracture (*n* = 20). All patients were followed for 24 weeks. Outcome was based on adverse events, fracture displacement on radiographs, fracture healing, range of motion, grip strength, pain, and the disability of the arm, shoulder and hand (DASH) score.

**Results:**

There were no serious adverse events in the study, but the pin tract infection rate was significantly lower in the Augment® group. There was no difference between the groups in fracture healing time, based on number of healed cortices or fracture displacement. The Augment® group had an early temporary significant decrease in wrist flexion, but no difference in range of motion at 24 weeks. There were no differences between the two treatment groups for any other outcome variables.

**Conclusion:**

rhPDGF-BB/β-TCP (Augment®) is safe and convenient for local administration into wrist fractures. In this pilot study, we could not detect any reduced healing time in the Augment® group although potential efficacy should be addressed in larger studies.

**Clinical trial registration number:**

The clinical trial registration number for the study protocol is BMPI-2014-02-E.

## Background

Platelet-derived growth factor (PDGF) has several functions in humans including promotion of angiogenesis and wound healing [1-3]. Both *in vitro* and *in vivo* preclinical studies have shown that recombinant human platelet-derived growth factor BB (rhPDGF-BB) stimulates bone formation [4,5]. It has also been demonstrated that application of rhPDGF-BB has a stimulatory effect on fracture healing in rabbit tibia fractures [6] as well as in tibial fracture models in rats [7-9]. So far, there are few clinical studies assessing the potential stimulating effect of PDGF on bone regeneration in humans. Local application of rhPDGF-BB gave a significant gain in bone formation in advanced periodontal osseous defects [10]. In studies on foot fusions, rhPDGF-BB was found to represent a safe and efficacious treatment alternative to autologous bone graft [11-13]. There are no available studies in humans where an rhPDGF-BB containing matrix has been used in acute fractures.

The primary aim of the present prospective randomized controlled feasibility study was to assess the safety and utility of rhPDGF-BB (Augment®) when applied locally in acute distal radius fractures. Our hypothesis was that local application of rhPDGF-BB (Augment®) into distal radius fractures is a safe and utile procedure.

## Methods

Forty consecutive patients aged 50 years or older, with a displaced unstable distal radius fracture, not suitable for conservative treatment, were included. All patients were treated at the Department of Orthopedics, Uppsala University Hospital, Sweden. Exclusion criteria were high-energy fractures, open or intraarticular displaced fractures, soft tissue infection at the operative site, previous wrist fracture on either side, bilateral fractures, fracture involving the shaft of the radius, or ulna other than a simple fracture through the styloid, patient currently undergoing radiotherapy or chemotherapy, patient with metabolic disorder or chronic medication known to adversely affect the skeleton, or physically or mentally compromised patients.

The study was approved by the local ethical committee of Uppsala University. All patients gave written informed consent before entering into the study.

After signing the informed consent form, each patient underwent treatment that included closed reduction and a bridging external fixation (Hoffman Compact II, Stryker AB, Malmö, Sweden) under general anesthesia. All patients were operated on by the authors. All patients were given a single dose of 2 g isoxazolyl penicillin before surgery as prophylaxis. Two Apex™ screws (APEX Fasteners Inc., Monrovia, CA, USA) were placed from the dorsal side into the radius and two Apex™ screws were placed from the dorso-radial side into the second metacarpal bone. After the external fixation had been mounted, the fracture was reduced under fluoroscopic guidance into an anatomic or near anatomic position after which the frame was locked. K-wires for temporary fixation were not used in any case, mainly because no severely displaced intraarticular fractures were included. The patients were then randomized in the operating room to one of two treatment groups using a closed envelope technique with strictly sealed, numbered, and not transparent envelopes.

The two treatment groups consisted of one group where in addition to the external fixation and closed reduction, Augment® (rhPDGF-BB/β-TCP) (BioMimetic Therapeutics Inc., Franklin, TN, USA) was applied locally into the fracture void while the controls were treated with external fixation alone. Twenty patients were allocated to each treatment group. In the Augment® group, the first ten patients were treated with Augment® Bone Graft, while the last ten patients were treated with Augment® Injectable. Augment® Bone Graft and Augment® Injectable are two separate formulations that combine rhPDGF-BB (0.3 mg/ml) with a synthetic bone matrix consisting of β-tricalcium phosphate (β-TCP). Augment® Injectable also contains soluble type I collagen (Kensey Nash Corp.; Exton, PA, USA), which makes it injectable through a canula.

In the Augment® group, a 1-cm dorsal incision over the fracture site was made. Using an elevator, the cancellous bone inside the fracture void was slightly impacted to allow for insertion of 5 ml of Augment® Bone Graft in the fracture void in the first cohort or 3 ml of Augment® Injectable in the last cohort. In both groups, the amount of rhPDGF-BB delivered to each patient was 0.3 mg. The skin was closed with a single suture and covered with sterile gauze. Pin sites were also covered with sterile gauze, similar in both treatment groups, with change of gauze twice a week.

Radiographs including AP and lateral views were taken postoperatively and at follow up at 1, 3, 6, 12, and 24 weeks of the injured wrist while radiographs of the uninjured wrist were taken for comparative reasons at 3 weeks. Bone mineral density was measured by DEXA (DPX-L, Lunar Co, Madison, WI, USA) on the uninjured wrist within 4 weeks. A research physiotherapist, who was not involved in the treatment of the patients, measured range of motion (ROM), grip strength, pin site condition, local signs of inflammation including redness and swelling, pain (VAS scale), and DASH-score according to a specific protocol at all visits. Active range of motion was measured in both wrists using a goniometer with the unaffected wrist used as the normal range for each patient. Grip strength was assessed based on the average of three measurements on each occasion using a Jamar dynamometer [14]. In addition, all patients were seen by another physiotherapist at the department directly after surgery, as well as on follow-up visits during the course of treatment, to get instructions related to their rehabilitation. Pin site condition was assessed twice a week by a nurse [15]. The external fixation was removed at 6 weeks in all patients.

All radiographs were examined by two independent assessors according to a predefined protocol that included measurement of the dorsal and radial angulation and axial compression. As the β-TCP granules in Augment® makes the product somewhat visible on radiographs, it was not possible to ensure blinding of the two assessors of the radiographs although they were blinded with respect to all clinical variables. Fracture healing was assessed at 6, 12, and 24 weeks. A fracture was defined as healed when three cortices were bridged by bone.

### Statistics

The independent *t*-test was used to compare the two groups for baseline characteristics and range of motion. Confidence interval (95%) was used to compare the radiographic results, pain, DASH, and grip strength. Chi square test was used for comparison of proportions, except for rate of pin infection where Fisher’s exact test was used. A *p* value less than 0.05 was considered as statistically significant.

## Results

There were no significant differences at enrollment between the two treatment groups with regard to age, gender, weight, height, smoking habits, working status, fractured side, hand dominance, or bone mineral density. Neither were there any differences between treatment groups regarding proportion of different fracture types (Table 1). The pre- and postoperative radiographs revealed no significant difference in fracture position between the two treatment groups. This means that the two groups had a very comparable starting point with respect not only to demographics but also to the radiological fracture variables.

**Table 1:** **Baseline characteristics (mean and standard deviation)**

	**Augment® (** ***n*** **= 20)**	**Controls (** ***n*** **= 20)**	***p***
Gender (M/F)	1/19	1/19	NS
Age (years)	65 ± 9.3	65 ± 8.2	NS
Length (cm)	166 ± 5.1	166 ± 7.4	NS
Weight (kg)	65 ± 10.2	71 ± 13.6	NS
Injured side (dx/sin)	8/12	6/14	NS
Injured side (dominant/nondominant)	8/12	6/14	NS
BMD (T-score intact wrist)	−1.66 ± 1.11	−1.14 ± 1.18	NS
Smoking (yes/no)	2/18	2/18	NS
Fracture classification (AO):			
23A3.2	3	3	NS
23A3.3	0	2	NS
23C2.1	7	5	NS
23C2.2	8	5	NS
23C3.2	2	5	NS
Time in ex fix, days (range)	42 (38–45)	42 (37–44)	NS

**Figure 1:**
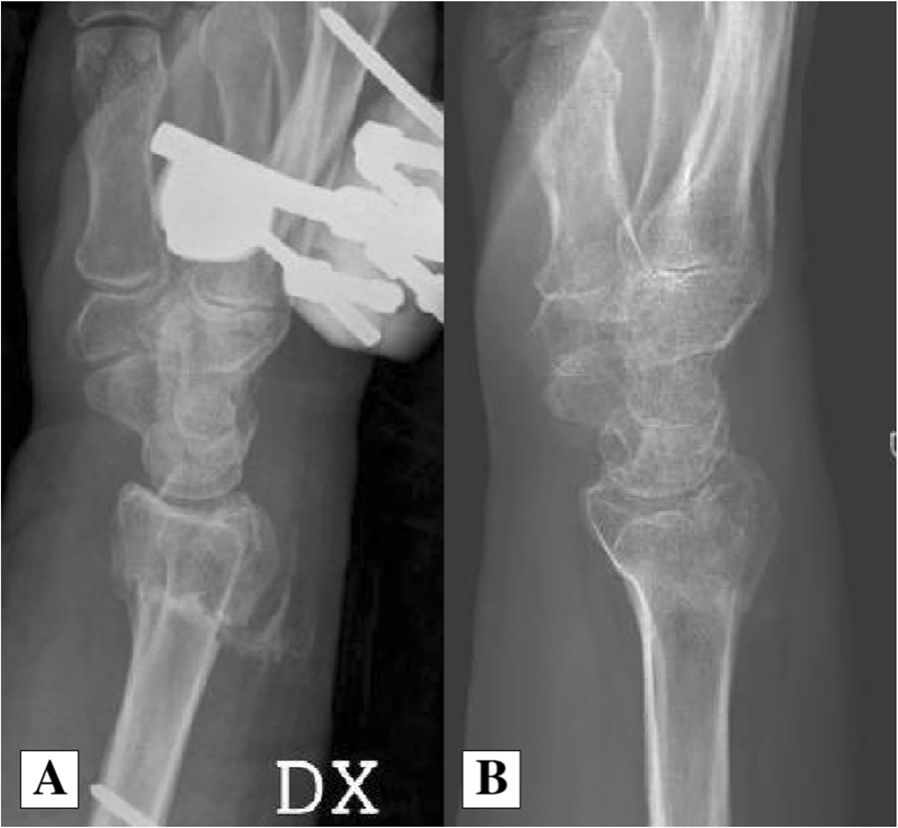
**Postoperative radiograph and 6 months after.** Postoperative radiograph after external fixation and dorsal injection of Augment® into the fracture gap **(A)**. At 6 months, all Augment® has resolved **(B)**.

All 40 patients completed the follow-up according to the protocol. There were no serious adverse events during the study period. In almost all patients in the Augment® group, the postoperative radiographs revealed small amounts of β-TCP particles in the soft tissue on the dorsal side. In Augment® Injectable patients, but not in Augment® Bone Graft patients, there was also leakage of material into the volar soft tissues as assessed on the postoperative radiographs. Almost all Augment®, whether contained in the fracture void or in the surrounding soft tissues, was resorbed at 24 weeks (Figure 1A,B).

There were three shoulder-hand-finger syndromes in the Augment® group and one in the control group, but this difference was not significant. All patients recovered following physiotherapy. No infections or signs of inflammation were observed at the fracture site in any of the Augment® patients. There were two pin infections in one patient in the Augment® group, while in the control group, pin infections occurred around 18/80 (22.5%) pins in ten patients (*p* = 0.0001 for pin infections and *p* = 0.017 for patients). No fixation pin had to be prematurely removed, and all pin-related infections healed uneventfully following oral antibiotics or pin removal at 6 weeks.

**Figure 2:**
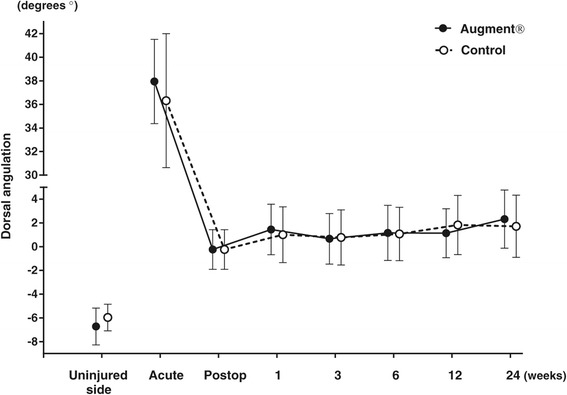
**Dorsal angulation on radiographs (mean with 95% CI).**

**Figure 3:**
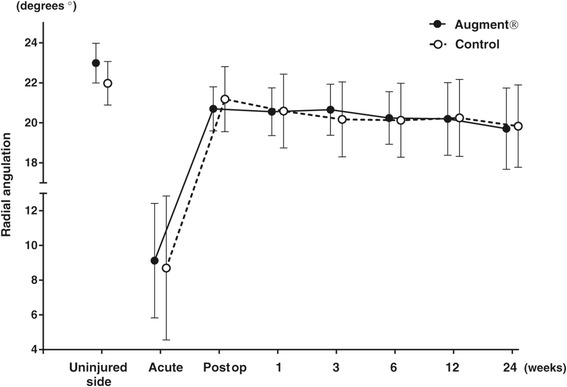
**Radial angulation on radiographs (mean with 95% CI).**

**Figure 4:**
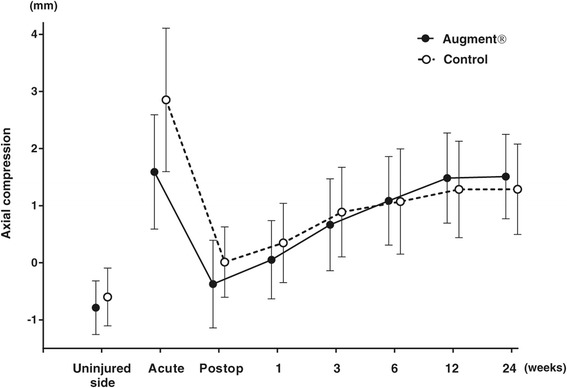
**Axial compression on radiographs (mean with 95% CI).**

All fractures healed during the period of follow-up. At 6 weeks, no fracture, in any of the two groups, was radiologically healed according to the criteria used in the study, i.e., three cortices with bridging bone. At 12 weeks, 9 fractures in the Augment® group and 13 fractures in the control group were radiologically healed, while all fractures were radiologically healed at the final follow-up at 24 weeks. There were no significant differences between the two groups with respect to fracture position at any follow-up visit or at the final follow-up at 24 weeks (Figures 2, 3 and 4).

Clinical assessment revealed that the patients treated with Augment® had significantly less wrist flexion at 6 and 12 weeks, but not at 24 weeks, compared to the control group, while there were no differences between the two treatment groups for any of the other wrist movements (Table 2).

**Table 2:** **Decrease in range of motion (degrees) of the radio carpal joint compared to uninjured side (mean and standard deviation)**

		**Augment®**	**Controls**	***p***
Wrist flexion	6 weeks	−46 ± 12	−36 ± 9	0.006
	12 weeks	−35 ± 11	−23 ± 14	0.007
	24 weeks	−24 ± 9	−16 ± 12	NS
Wrist extension	6 weeks	−57 ± 11	−50 ± 17	NS
	12 weeks	−22 ± 14	−16 ± 12	NS
	24 weeks	−10 ± 11	−9 ± 8	NS
Radial deviation	6 weeks	−23 ± 8	−22 ± 10	NS
	12 weeks	−12 ± 8	−8 ± 9	NS
	24 weeks	−6 ± 6	−3 ± 9	NS
Ulnar deviation	6 weeks	−23 ± 9	−18 ± 8	NS
	12 weeks	−16 ± 9	−12 ± 9	NS
	24 weeks	−11 ± 8	−8 ± 8	NS
Supination	1 weeks	−33 ± 20	−31 ± 19	NS
	3 weeks	−39 ± 21	−36 ± 22	NS
	6 weeks	−43 ± 21	−37 ± 22	NS
	12 weeks	−24 ± 18	−21 ± 16	NS
	24 weeks	−8 ± 8	−9 ± 8	NS
Pronation	1 weeks	−27 ± 18	−24 ± 15	NS
	3 weeks	−24 ± 19	−24 ± 14	NS
	6 weeks	−16 ± 15	−12 ± 11	NS
	12 weeks	−11 ± 11	−4 ± 8	NS
	24 weeks	−4 ± 6	−3 ± 7	NS

**Figure 5:**
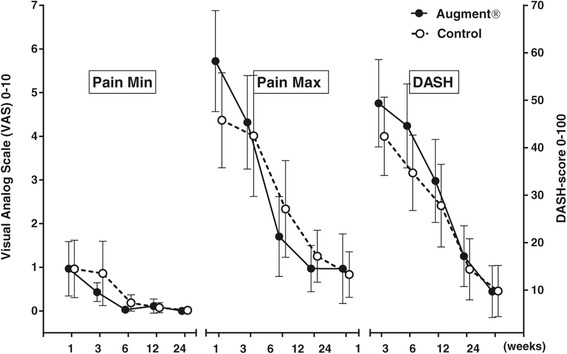
**Minimum and maximum pain, and DASH score (mean with 95% CI).**

**Figure 6:**
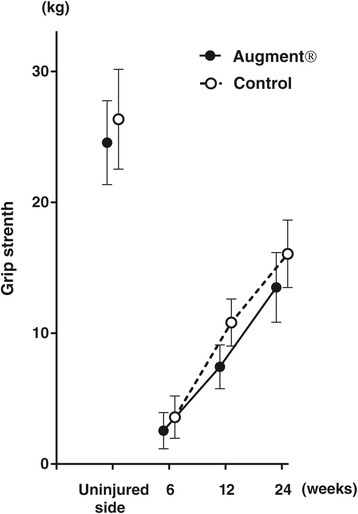
**Grip strength (mean with 95% CI).**

No statistically significant differences in DASH score or pain were found between the two treatment groups at any time point. DASH score decreased in both groups over time, and at 6 months, the average DASH score was 6.8 in the Augment® group and 6.6 in the control group (Figure 5). There was no significant difference in grip strength between the treatment groups at any time point. At 24 weeks, grip strength was still reduced on average 41% for Augment® patients and 39% for controls (about 10 kg in absolute value) when compared to the uninjured side (Figure 6).

The operating time was longer in the Augment® group, 42 versus 29 min, when compared to the control group (*p* < 0.0001), i.e., application of Augment® added on average 13 min to the procedure, with no significant difference between Augment® Bone Graft or Augment® Injectable.

## Discussion

The main findings of this study were that all fractures healed both radiographically and clinically without differences between the treatment groups, there were no serious adverse events in any of the patients, and Augment® (rhPDGF-BB/β-TCP) could be handled and applied locally at the fracture site as intended. From a handling perspective, Augment® Injectable was relatively easier to use as it could be injected into the fracture void compared to Augment® Bone Graft, where the technique used for application had to be adjusted to allow adequate placement of the compound containing β-TCP particles. The study was a pilot clinical trial and therefore not powered to measure efficacy. With the present number of patients, the observed standard deviation and a statistical power of 80%, an average difference of 4° in dorsal angulation between the groups could have been detected. A difference of 4° in dorsal angulation can be challenged as to whether or not it is of clinical importance. Accordingly, it had been possible to detect a clinical significant difference in dorsal angulation.

In animal studies, as well as in periodontal bony defects in humans, it has been shown that recombinant PDGF accelerates bone formation when compared to controls [6-10]. In the present study, it was not possible to detect any difference in bone formation over time between the two treatment groups. There are several potential reasons for this discrepancy. The distal radius fracture was chosen in part because it is a common fracture and because external fixation is a viable option for stabilization, even though plate fixation has gained in popularity over the last years. By using external fixation, there was no metal at the fracture site, thereby assessment of radiological healing could be done without disturbance of hardware. In addition, fracture stability after frame removal could be measured and used as an indirect sign of the mechanical competence of the callus tissue. On the other hand, a distal radius fracture is located in the metaphysis which consists of spongious bone with high healing potential due to extensive vascularization. A distal radius fracture usually heals rapidly, and delayed union is very unusual. One could therefore argue that a metaphyseal fracture, like the distal radius fracture, already has such a high healing rate that it hardly can be further accelerated. Based on this, fractures in the diaphyseal bone would be more appropriate when efficacy of Augment® is investigated.

Furthermore, conventional radiography is a fairly blunt tool for quantitative assessment of new bone formation in a metaphyseal area, and the presence of calcium phosphate particles in the product implanted at the fracture site might be difficult to distinguish from the bone. In addition, since only a limited number of radiographic examinations can be allowed within a clinical study, a subtle change in bone formation over time is difficult to observe, and therefore, it is not possible to detect a small difference in bone formation between the two groups. Before injecting Augment® into the fracture gap, a small void was created at the fracture site by impacting the spongious bone inside the fracture with an elevator. This impaction created less bone volume inside the cavity, and more dense bone in the margins of the void, which, together with calcium phosphate particles, made it more difficult to assess the healing of the fractures in the Augment® group. The impaction of bone could also have increased the instability and delayed the bone healing in the Augment® group. All fractures in the study were treated with external fixation for 6 weeks. It is a common duration of treatment for distal radius fractures and often leads to advanced healing without severe displacement of the fracture after removal of the fixation. We chose 6 weeks of treatment in order not to compromise the position of the fractures, but by choosing such a long fixation time, a potential acceleration of healing and improved early fracture stability in the Augment® group could have been missed.

The impaired wrist flexion in the Augment® group was an unexpected finding that we believe was caused by the dorsal incision used to get access to the fracture for implanting the material and not an effect of the Augment® *per se*. Despite that the incision was small, local scar tissue and adhesions in the soft tissue on the dorsal side of the wrist seem as the most plausible cause for the reduced flexion. This can explain the observed restriction in motion that involved only flexion, while wrist motion in other directions did not differ between the treatment groups. If the restricted movement had been caused by the Augment®, it seems reasonable that some local soft tissue reaction would have been visible. In comparison, when implanting bone morphogenetic protein (BMP), it is known that the implanted substance induces a local inflammatory response that can include swelling of clinical importance [16-18]. In contrast to the adverse effects reported for BMP, there were no signs of inflammation in any of the Augment® patients in the present study.

The finding with significantly fewer pin site infections in the Augment® group, 2/80 versus 18/80 in the control group, was surprising and no reasonable explanation has been identified. All factors that are known to reduce the risk for pin site infections were kept constant in all patients, which make it unlikely that this finding was due to a systematic error in the care or evaluation of the pin sites. In previous studies, local application of a PDGF-BB containing dressing to diabetic ulcers in the lower extremity have been shown to significantly increase the incidence of complete wound closure and time to healing [19], which is an expected outcome based upon the biologic mode of action of rhPDGF-BB. As Augment®, in the present study, was administered at the fracture site, and not at the pin site, there is no obvious explanation to be found in a local soft tissue effect by Augment®. The decrease in pin site infections might instead be due to a reason not directly related to the effect of PDGF-BB. For instance, a reduction in finger movement and forearm rotation caused by pain from the dorsal incision at the administration site in the Augment® group could have had a protective effect against pin infection since less soft tissue motion around the pins will reduce the irritation in the surrounding soft tissue. In all, the mechanism of a potential protective action against pin infection by Augment® is unclear, although not inconsistent with the established wound healing capacity of rhPDGF-BB. In order to reveal a better understanding, this question should be addressed in a study with pin site infection being the primary outcome.

## Conclusions

rhPDGF-BB/β-TCP (Augment®) is safe and convenient for local administration into wrist fractures. In this pilot study, we could not detect any reduced healing time in the Augment® group although potential efficacy should be addressed in larger studies.
